# Editorial: Herbal medical products and natural products targeting aging and age-related disorders--ethnopharmacological perspectives

**DOI:** 10.3389/fphar.2024.1368389

**Published:** 2024-01-31

**Authors:** Hammad Ullah, Haroon Khan, Miguel Angel Prieto Lage, Maria Daglia

**Affiliations:** ^1^ Department of Pharmacy, University of Napoli Federico II, Naples, Italy; ^2^ Department of Pharmacy, Abdul Wali Khan University Mardan, Mardan, Pakistan; ^3^ Universidade de Vigo, Nutrition and Bromatology Group, Department of Analytical Chemistry and Food Science, Instituto de Agroecoloxía e Alimentaci on (IAA)–CITEXVI, Vigo, Spain; ^4^ Centro de Investigação de Montanha (CIMO), Instituto Politécnico de Bragança, Bragança, Portugal; ^5^ International Research Center for Food Nutrition and Safety, Jiangsu University, Zhenjiang, China

**Keywords:** natural products, herbal medicines (HM), aging, age-related disorders, prevention

## Introduction

The population aged ≥60 years is growing worldwide. The World Health Organization (WHO) reported that the elderly population is expected to increase from 1 billion in 2020 to 1.4 billion in 2030 and 2.1 billion in 2050 (World Health Organization, 2022). This increase in life expectancy around the globe is accompanied with the enhancing frequency of aging-associated disorders, mainly due to the deterioration of metabolic, circulatory, and immune functioning. Inflammaging (chronic low-grade inflammation) is considered as one of the main drivers of the initiation and progression of diseases such as sarcopenia, osteoporosis, metabolic ailments, atherosclerosis, neurodegenerations, and carcinogenesis (Ullah et al., 2022). However, the actual etiology of these disorders is complicated due to the complex interaction of genes and environment, making their management more challenging in real-world circumstances. Currently, pharmacological interventions remained the mainstream option for the treatment of age-related diseases although drugs are associated with increased risk of the occurrence of adverse effects due to the increased susceptibility of adult population to drug effects, altered pharmacokinetic parameters, and use of polypharmacy, which may also increase the likelihood of drug interactions (Baldoni et al., 2010; Goldberg et al., 2022; Kwak et al., 2022).

Natural products from terrestrial and marine sources have been of great interest in the field of aging and age-related disorders due to their potential health-promoting properties. Extensive literature data have reported a wide range of natural products for the prevention and/or treatment of most challenging diseases. While the research in this area is ongoing, some natural products have shown promising properties in contributing to healthy aging and potentially mitigating age-related disorders. It is important to note that the effectiveness of these products may vary, and more research is needed to establish their therapeutic benefits (Cătană et al., 2018; Bjørklund et al., 2022). In this context the current Research Topic “Herbal medical products and natural products targeting aging and age-related disorders–ethnopharmacological perspectives” is designed to focus on the preclinical and clinical research updates on the chemically-characterized botanical extracts, derived phytochemicals, and food bioactive ingredients targeting aging and age-related disorders (such as metabolic, cardiovascular, neurological, and bone diseases, and cancer), and their mechanistic targets with reference to age-related disorders including cellular aging signaling. The main aim of this Research Topic was to identify natural products with health-span and longevity-promoting effects.

A total of six articles including two original research (Xu et al. and Subramanian et al.) and four literature reviews (Gao et al., Chen et al., Jing et al., and Ma et al.) were published. Xu et al. investigated the effects of Chinese traditional herb (*T. pericarpium*) in improving microcirculation in patients with acute myocardial infarction following percutaneous coronary intervention. The results of this study demonstrated significant improvement in coronary microcirculation in *Trichosanthes pericarpium* treated patients as suggested by the decrease in corrected thrombolysis in myocardial infarction frame count (CTFC), index of microcirculatory resistance (IMR), and major adverse cardiac events, with an improvement in left ventricular ejection fraction and left ventricular internal dimension. While evaluating the neuroprotective potential of *M. quadrifolia* Linn. in rodents, Subramanian et al. observed the amelioration of monosodium glutamate-induced excitotoxicity in rats with quercetin-enriched *Marsilea quadrifolia* extract. The main effects included the reduced locomotor score and impaired memory/learning, increased blood levels of sodium and calcium, and less neuronal disorganization along with cerebral edema and neuronal degeneration.

Gao et al. reviewed and summarized the available literature on the antiaging potential of polyphenols (quercetin, luteolin, catechins, resveratrol, curcumin, and lignans), saponins, alkaloids, and polysaccharides. The cellular and molecular targets mediating the longevity-extending effects of these products include sirtuin, AMP-activated protein kinase (AMPK), mammalian target of rapamycin (mTOR), p53, and insulin/insulin-like growth factor-1 signaling pathways. Chen et al. reviewed a literature to highlight the protective effects of natural coumarin osthole against osteoporosis. It was reported that osthole may enhance the osteoblast-related bone formation with a decreased osteoclast-related bone resorption and suppressed osteoporosis-related fragility fracture. The mechanistic targets of osthole may include activation of Wnt/β-catenin and BMP-2/Smad1/5/8 signaling pathways to promote osteogenic differentiation and suppressions of RANKL-induced osteoclast activity. Jing et al. discussed the natural products modulating the crosstalk between gut microbiota and immune response in atherosclerosis, as it has been reported over the past decades that gut microbiota and its metabolites regulate the functional expression of immune cells, possessing a considerable impact on the progression of atherosclerosis. Natural products such as polyphenols, alkaloids, and saponins are found to modulate the relative abundance of gut microbiota, which may result in delaying the progression of atherosclerosis, suppressing the monocytes/macrophages migration, downstream regulation of inflammatory mediators, regulation of Treg/Th17 balance, and suppressing the foam cells formation. Ma et al. provided a comprehensive review on the Tibetan medicine Shengdeng (rich in flavonoids and triterpenoids) including their traditional uses and reported pharmacological uses including antioxidant, anti-inflammatory, antimicrobial, anti-arthritic, and anti-cancer activities.

In conclusion, this Research Topic collects articles on herbal extracts and isolated compounds with potential antiaging effects, supporting the fact that natural products may serve as a sustainable source of chemical entities promoting healthy aging and counteracting age-related diseases. However, it is essential to approach the use of natural products with caution and after consult with qualified healthcare professionals before incorporating them into a routine practice, particularly in the context of managing age-related disorders. Moreover, pharmacokinetic and toxicity studies should be conducted to further establish the efficacy and safety of these products.
